# Breast cancer awareness, risk factors and screening practices among future health professionals in Ghana: A cross-sectional study

**DOI:** 10.1371/journal.pone.0253373

**Published:** 2021-06-24

**Authors:** Sandra Osei-Afriyie, Albert Kwesi Addae, Samuel Oppong, Hubert Amu, Emmanuel Ampofo, Eric Osei

**Affiliations:** 1 Department of Physician Assistantship, School of Medicine, University of Health and Allied Sciences, Ho, Ghana; 2 Department of Psychological Medicine and Mental Health, School of Medicine, University of Health and Allied Sciences, Ho, Ghana; 3 Department of Epidemiology and Biostatistics, School of Public Health, University of Health and Allied Sciences, Ho, Ghana; 4 Department of Population and Behavioural Sciences, School of Public Health, University of Health and Allied Sciences, Ho, Ghana; 5 Directorate of Human Resource, University of Cape Coast, Cape Coast, Ghana; 6 Department of Public Health Graduate School, Yonsei University, Seoul, Republic of Korea; University of Insubria, ITALY

## Abstract

**Background:**

Like many other women in the developing world, the practice of breast cancer screening among Ghanaian women is unsatisfactory. As a result, many cases are diagnosed at advanced stages leading to poor outcomes including mortalities. An understanding of the awareness and predictors of breast examination is an important first step that may guide the design of interventions aimed at raising awareness across the general population. This study aimed to explore the awareness, risk factors, and self-reported screening practices of breast cancer among female undergraduate students at the University of Health and Allied Sciences.

**Methods:**

This cross-sectional study was conducted among 385 female undergraduate students using a pre-tested questionnaire. Data were analysed using Stata Version 13.1 and presented using descriptive and inferential statistics comprising frequency, percentage, chi-square, and binary logistic regression. Odds ratios and 95% confidence intervals were computed to quantify the association between regular Breast-Self Examination (BSE) and socio-demographic characteristics of respondents.

**Results:**

Seventy-three per cent of the students were aware of breast cancer, with social media being the most important source of information (64.4%). The prevalence of breast cancer risk factors varied from 1% of having a personal history of breast cancer to 14.3% for positive family history of breast cancer. Current use of oral pills/injectable contraceptives was confirmed by 13.2% of participants; 20% were current alcohol users and10.1% were physically inactive. Regarding breast examination, 42.6% performed BSE; 10.1% had Clinical Breast Examination (CBE), while 2.3% had undergone mammography in the three years preceding the study. Women who did not believe to be susceptible to breast cancer (AOR: 0.04; 95%CI: 0.02–0.09) and those who did not know their risk status (AOR: 0.02; 95%CI: 0.005–0.57) were less likely to perform regular BSE compared to those who displayed pessimism. Further, women with no religious affiliation had 0.11 (95%CI: 0.02–0.55) odds of examining their breast regularly compared to Christians.

**Conclusion:**

This study demonstrated moderate awareness of the modalities of breast cancer screening and the risk factors of breast cancer among the students. However, there exists a gap between awareness and practice of breast cancer screening, which was influenced by optimism in breast cancer risk perception and religion. Awareness campaigns and education should be intensified in the University to bridge this gap.

## Background

Breast cancer is the most prevalent cancer in women and generally the second most common cancer globally, with approximately 1.4 million cases diagnosed annually [1]. In 2018, 2.1 million incident cases of breast cancer were diagnosed worldwide (second most common cancer overall after lung cancer) representing nearly 12% of all incident cancer cases and an estimated 627,000 deaths were expected to occur globally [2, 3]. Generally, the breast cancer rate is higher in the developed world than the developing countries, which may be as a result of certain lifestyles and reproductive factors that are more common in the developed world. The difference may be exaggerated due to relatively low awareness, screening practices, and diagnoses in the developing countries, though the rates are increasing rapidly in many developing countries [4].

In Ghana, breast cancer is becoming a great public health challenge among women. With about 2,900 incident cases occurring annually, and one-eighth of them dying from it, the disease has become the most common cancer-related death among Ghanaian women [5, 6]. Previous studies have revealed an increased breast cancer burden in Ghana over the past decade. Clegg-Lamptey et al. [7] reported in 2009 that breast cancer now accounts for about 16% of all cancers and the most common cancer female cancer in Ghana. Naku et al. [8] estimated the incidence of breast cancer to be 76 per 100 000 Ghanaian women.

It is anticipated that the incidence of breast cancer will increase as Ghana’s population ages and women adopt western lifestyles [9]. Studies have shown that breast cancer is increasingly becoming common among younger Ghanaian women, and they present at a more advanced stage of the disease [5, 7, 10]. The advance stage at diagnosis is due to patients’ delay in seeking healthcare, which can be up to 10 months after the onset of symptoms, as a result of lack of awareness of the disease at its early stage and stigma associated with the disease [8, 11, 12].

Like most cancers, the primary risk factor for breast cancer in women is older age. Many other risk factors alter the exposure of breast tissues to reproductive hormones [4]. Some of these are modifiable, and include weight gain or being overweight/obese, long term use of the postmenopausal hormone, alcohol consumption, and physical inactivity [4, 13]. Long menstrual history (younger age at menstruation and/or end later age at onset of menopause), never given birth, having one’s first child after age 30, and current use of hormonal contraceptives are other reproductive factors that influence the risk of breast cancer [11, 12]. Personal medical or family history of breast cancer are also known to increase one’s risk for breast cancer [4].

Early diagnosis of breast cancer can increase the chance of early case detection and favourable outcomes, resulting in improved survival rates and quality of life of women and is therefore important public health strategy at all settings [2]. However, studies have demonstrated that factors related to women´s awareness, knowledge and perceptions about the disease may contribute significantly to health-seeking behaviours [14, 15].

Notwithstanding that mammography is known to be the most effective screening tool for early detection of breast cancer [16], Breast-Self Examination (BSE) may be useful in resource-limited countries to detect any abnormalities in the breast as it provides an opportunity for women to be familiar with their breasts and promptly report any changes [17]. Additionally, clinical encounters by women provide the opportunity for women to have a number of important clinical activities such as breast cancer risk assessment, education about lifestyle, counselling, and clinical breast examination (CBE) that may not otherwise be done [17]. There are no current national breast cancer screening protocols in Ghana or countrywide literacy initiatives, and there is limited availability of mammography or ultrasound machines [5]. Although the effectiveness of BSE and CBE in detecting breast cancer is debatable [16], they have been campaigned as major screening tools given their availability at little to no cost among limited-resource settings in most developing countries.

Women who practice regular breast screening have been known to have a lower risk of developing an advanced form of breast cancer than those who do not. Reports have shown low uptake of breast cancer screening among women in many sub-Saharan African countries and this results in late detection and increased mortality in affected women [18].

Even though the incidence and mortality of breast cancer have been on the increase, there is a paucity of the empirical literature on student’s awareness, risk factors and screening practices in Ghana. This population is especially important because they have abundant access to health information as health students and they are potential sources of information for non-health students and the general population. College students have a significant influence on colleague students and also, findings regarding this target group have implications for their capacity in the role of promoting screening for breast cancer as potential health professionals. This study, therefore, explored breast cancer awareness, selected risk factors, and screening practices among female undergraduate students, to provide information for the control, prevention, and early treatment of the disease.

## Materials and methods

### Study site and design

We conducted a cross-sectional study among female undergraduate students at the University of Health and Allied Sciences (UHAS), Ghana in February 2019. Ghana has a projected population of 30,280,482 in 275 districts distributed among 16 administrative regions as of June 2019 and is divided into some 75 ethnic groups with the Akans forming the majority. UHAS is among the newest public Universities in Ghana which was established in 2011 and is located at Ho in the Volta Region. UHAS is so far the only public University solely dedicated to the training of varied health professionals such as medical doctors, nurses, midwives, pharmacists, public health professionals etc. in Ghana. The University has two main campuses; the main campus, which is located at Ho (where this study was carried out), and the Hohoe campus, which has the School of Public Health. There are currently six schools/colleges in the University, with a current student population of about 3,752. Among the programmes offered by the University are Medicine, Physician Assistantship, Nursing, Midwifery, Physiotherapy, Pharmacy, Dietetics, Speech and Language Therapy, and Public Health [19].

### Sample size determination

We obtained the required sample size for this study using Cochran’s single proportion formula; n = z^2^ pq/d^2^, where n = sample size, z = z-score at 95% confidence level, p = estimated proportion of an attribute that is present in the population, q = 1-p, d = margin of error. This study assumed a margin of error(d) of 0.05 at 95% confidence level and an estimated proportion of 60.9% women with regular breast screening practice [20]. Adjusting for a non-response rate of 5%, 385 students were sampled and participated in the study. Thus, we sampled 385 students of which all participated.

### Sampling procedure

We used two levels of stratified sampling method to select the study participants from to ensure representativeness. First, students were stratified according to faculty (school), defined as one of the 5 constituent schools in the University, namely, School of Allied Health Sciences; School of Basic and Biomedical Sciences; School of Medicine; School of Nursing and Midwifery; and School of Pharmacy. For each faculty, students were stratified according to their level (year) of study, where simple random sampling was used to select the required number of students separately from each faculty and academic year. This was done by obtaining names of all female students according to their faculty and level of study from the University’s administration. The names of eligible students were arranged and numbered alphabetically separately for each faculty and level in a Microsoft excel sheet, where random numbers were generated, and the corresponding students selected to take part of the study. The number of students selected from each academic year and faculty was proportional to the students’ population. Selected students were approached by the lead author who was also a student in the University in January 2019 and after explaining the study’s objectives and procedures to them, all (100%) agreed to participate in the study. Students who were under 18 years of age were excluded from this study.

### Data collection tools and procedure

A structured pre-tested questionnaire was used to collect data from participants. The questionnaire contained closed-ended questions adapted from previously published studies that adapted validated questions and had the following sections: demographic information, breast screening practices, risk factors of breast cancer (Behavioural, reproductive, and hormonal factors), and awareness of breast cancer.

Consenting participants were handed printed copies of the questionnaire and were given 24 hours to fill their responses and return them anonymously to the researchers. The objectives of the study were explained to all participants before the questionnaires were given out. Filled questionnaires were checked daily by the researchers for consistency and omissions before collection. Where there were inconsistencies, participants were made to correct them in the presence of the researchers before they were taken.

#### Definition of variables

Table 1 presents the definitions of some of the study variables measured in this study.

**Table 1:** Definitions of key study variables used.

Variable	Definition	Reference
Breast cancer awareness	Having heard of Breast cancer from any source	
Breast cancer screening practice	Self-reported application of at least one of the following: Breast-self Examination, Clinical-Breast Examination, or Mammography	
Physical activity	Achieving 600 or more MET-minutes per week	[21]
Perceived risk of breast cancer	Responding “yes” to “are you at risk of Breast cancer” question	
Regular BSE	Performing Breast-Self Examination at least once per month	
Current alcohol user	Daily consumption of at least 1 standard drink	[22]
Family history of breast cancer	Any relative grandmother, mother, auntie, or sister ever diagnosed with Breast cancer	

### Data analysis

All analyses were carried out using STATA (Stata Corp, College Station) statistical package Version 13. Descriptive statistics were used to describe the study population in relation to relevant variables. Bivariable and multivariable logistic regression models were used to identify significant predictors of regular BSE defined as having examined the breast at least once every month. First, the association of regular BSE with each variable of interest (Participant’s age, religion, ethnicity, faculty of study, academic year, family history of breast cancer, having known or seen a breast cancer patient, perceived risk for breast cancer, physical activity, current Alcohol use and current use of contraception) was examined. Second, variables with p-value <0.20 in the first model were considered for inclusion to construct a model with risk factors independently associated with the outcome variable. The degree of association between dependent and independent variables was assessed using odds ratios (OR) with 95% confidence intervals (CI).

### Ethical issues

The study approval by the University of Health and Allied Sciences’ Ethics Review Committee with reference UHAS-REC A.8 [56] 18–19. Written informed consent was obtained from each respondent after explaining the study’s procedure and potential risk and benefits to them. Participants’ identifiers such as name and address were not collected.

## Results

### General characteristics of respondents

All 385 participants returned their completed questionnaire. The mean age of the study population was 22 ± 2.78. The majority (55.8%) were between 20 and 24 years old, while 1.3% were 30 years and above; 16.4% were currently married and 83.1% were single; 74.0% were Christians and 18.9% were Muslims. Regarding ethnicity, Akans were the majority (38.4%), followed by Ewes (30.9%), while 14.3% belonged to the Ga-Dangbe ethnic group. Respondents constituting 26.2% (n = 101) were in their first year of study, 21.8% were in the second year, 30.2% in the third year and 21.8% were fourth-year students. The comparative majority (52.5%) were studying Nursing and midwifery, 22.3% were Medical students, and 19.7% were Allied Health Sciences students (Table 2).

**Table 2:** General characteristics of respondents.

Variable	Frequency (N = 385)	%
**Age group (years)**		
<20	105	27.3
20–24	215	55.8
25–29	60	15.6
30+	5	1.3
**Marital status**		
Currently married	63	16.4
Divorced/Widowed	2	0.5
Never married	320	83.1
**Religion**		
Christian	285	74.0
Muslim	73	18.9
Traditionalist	1	0.3
No religion	26	6.8
**Ethnicity**		
Akan	148	38.4
Ewe	119	30.9
Ga-Dangbe	55	14.3
Others	63	16.4
**Academic year**		
First-year	101	26.2
Second-year	84	21.8
Third-year	116	30.1
Fourth-year	84	21.8
**Faculty**		
Allied Health Sciences	76	19.7
Basic and Biomedical Sciences	7	1.8
Medicine	86	22.3
Nursing & Midwifery	202	52.5
Pharmacy	14	3.6

### Breast cancer awareness and risk perception

Regarding breast cancer awareness, 281 (73.0%) of the respondents reported having ever heard of breast cancer. The remaining items in Table 2 were asked of the 281 women who had heard of breast cancer. The social media remained the most important source of information on breast cancer (181;64.4%), followed by teachers (173; 62%) and the electronic media (172; 61%). As presented in Table 2, the majority (200; 71.1%) of respondents were aware of mammography as a screening method for breast cancer; 196 (69.8%) were aware of BSE; while 28 (10.0%) of them did not know of any the screening methods. Family history of breast cancer (n = 236, 83.9%), genetics (n = 229, 81.7%) female sex (n = 173, 71.9%) and individual lifestyle (n = 176, 62.6%) were the most frequently indexed risk factors for breast cancer. Meanwhile, nulliparity (n = 100, 35.6%), early menses/menopause (n = 116,41.3%), and Obesity (n = 159, 56.6%) were the least known risk factors. Putting money in the brassiere was implicated as a potential risk factor for breast cancer by more than a third (n = 106, 37.7%) of study participants. The most common presentation of breast cancer aware of was a lump in the breast (n = 259, 92.2%), followed by nipple discharge (n = 219, 77.9%), and lymph node in the armpit (n = 191, 68%) while pulling off a nipple (n = 47, 16.7%), nipple itch (n = 55, 19.6), and swollen nipple (n = 97, 34.5) were the least known sign/symptom of breast cancer.

Among students who were aware of breast cancer, 129 (45.9%) thought that they do not have the chance of getting breast cancer, while 46 (16.4%) did not know whether they were at risk or not (Table 3).

**Table 3:** Breast cancer awareness and risk perception.

Variable	Frequency	%
**Have heard of breast cancer**		
Yes	281	73.0
No	104	27.0
Source of breast cancer information*		
Social media	181	64.4
Teacher	173	61.6
Electronic media	172	61.0
Friends and relatives	111	39.5
Health worker	101	35.9
Print media	45	16.0
Breast cancer screening method aware of*		
Breast Self-Examination	196	69.8
Clinical Breast Examination	144	51.2
Mammography	200	71.1
None	28	10.0
Awareness of breast cancer risk factors*		
Genetics	229	81.7
Drugs	69	24.6
Ageing	178	63.4
Female sex	202	71.9
Putting money in brassiere	106	37.7
Lifestyle	176	62.6
Nulliparity	100	35.6
Early menses/late menopause	116	41.3
Obesity	159	56.6
Family history of breast cancer	236	83.9
None	14	5.0
Awareness of signs/symptoms*		
Lump in breast	259	92.2
Nipple discharge	219	77.9
Swollen nipple	97	34.5
Ulcerated breast	136	48.4
Inverted nipple	148	52.7
Pain in breast	151	54.1
Redness of breast	102	36.3
Nipple itch	55	19.6
Lymph node in the armpit	191	68.0
Pulling in of the nipple	47	16.7
**Breast cancer risk perception**		
At risk	106	37.7
Not at risk	129	45.9
Don’t know	46	16.4

*Multiple responses.

### Breast cancer risk factors

Among the 385 study participants, menarche occurred below age 12 in 48 (12.5%) of them, while 72 (18.7%) were more than 14 years at menarche. The prevalence of current oral pill/injectable contraceptive use was 13.2% (n = 51). The occurrence of breast cancer in the family was confirmed in 55 (14.3%) of the respondents; 14 (4%) had their first-degree relatives (Mother, Sister) affected with the disease, while 41 (11%) were second-degree relatives. Four (1%) of the students had a personal history of breast cancer. Regarding behavioural factors, 39 (10.1%) did not engage in physical activity and 77 (20%) currently use at least one standard of alcohol per day (Table 4).

**Table 4:** Selected breast cancer risk factors.

Variable	Frequency	%
**Age at menarche (years)**		
<12	48	12.5
12–14	265	68.8
≥15	72	18.7
**Current use of oral pills/injectables contraceptive**		
Yes	51	13.2
No	334	86.8
**Positive family history of breast cancer**		
Yes	55	14.3
No	330	85.7
**Relative with breast cancer (N = 55)**		
Sister	2	3.6
Mother	12	21.8
Grandmother	29	52.7
Auntie	12	21.8
**Personal history of breast cancer**		
Yes	4	1.0
No	381	99.0
**Physical activity**		
Active	346	89.9
Inactive	39	10.1
**Current alcohol consumption (drink/day)**		
0	308	80.0
≥1	77	20.0

### Breast cancer screening practices

Of the 385 participants, 212 (55.1%) ever applied at least one breast cancer screening method. Of these, 164 (42.6%) practised BSE, 39(10.2%) had undergone CBE, and 9 (2.3%) were screened for breast cancer via mammography. Of those who performed BSE, 136 (82.9%) did it at least once in every month; 6 (3.7%) examine their breast yearly, and 22 (13.4%) did it at random; 115 (70.1%) of them learned BSE skills from their school-teachers, 112 (68.3%) from the media, 37 (22.6%) were taught by their friends, and 26 (15.9%) learned it from their mothers. The most common (28.1%) reason for not practising BSE was “1 do not know how to perform it” followed by “I have no family history of breast cancer” (23.1%) and “I am not at risk of breast cancer” (17.2%), while 63 (28.5%) had no reason for not doing BSE (Table 5).

**Table 5:** Breast cancer screening practices.

Variable	n	%
**Breast cancer screening method ever used**		
Breast Self-Examination	164	42.6
Clinical Breast Examination	39	10.1
Mammography	9	2.3
Never screened	173	44.9
**Frequency of BSE (N = 164)**		
More than once a month	38	23.2
Once a month	98	59.8
Once a yearly	6	3.7
At random	22	13.4
Source of BSE skills*		
Mother	26	15.9
Teacher	115	70.1
Media	112	68.3
Friend	37	22.6
**Reason for not doing BSE (N = 221)**		
Do not know how to do it	62	28.1
Not necessary	3	1.3
Do not have family history of breast cancer	51	23.1
Not at risk of breast cancer	38	17.2
Do not have time	4	1.8
No reason	63	28.5

*Multiple responses applied.

### Predictors of breast self-examination

Table 6 shows the results of bivariable and multivariable logistic regression analyses aimed at identifying variables associated with the odds of performing regular BSE. After adjusting for confounding effect of the variables, women who were between 25 and 29 years old were 5.13 (95%CI: 1.18–22.26; P = 0.03) times more likely to perform regular BSE compared to those less than 20 years. Further, women with no religious affiliation had 60% less odds of performing regular BSE compared to Christians. Women who expressed optimism regarding breast cancer risk (AOR: 0.04; 95%CI: 0.02–0.09; p<0.001) and those who did not know their risk level (AOR: 0.02; 95%CI: 0.005–0.57; p<0.001) were less likely to perform regular BSE compared to those who were pessimistic about breast cancer risk.

**Table 6:** Logistic regression analysis of predictors of regular breast self-examination for breast cancer.

Characteristic	Regular BSE (N = 136)	Irregular BSE (N = 249)	cOR (95%CI)	aOR (95%CI)	P-value
	n(%)	n(%)			
**Age group**					
< 20	14(13.3)	91(86.7)	1	1	
20–24	83(38.6)	132 (61.4)	4.09 (2.18–7.64)	1.89 (0.59–6.07)	0.28
25–29	35(58.3)	25(41.7)	9.1 (4.25–19.48)	5.13 (1.18–22.26)	0.03
30+	4(80.0)	1(20.0)	26.0 (2.70–249.76)	4.49 (0.30–67.38)	0.28
**Academic year**					
First-year	11(10.9)	90(89.1)	1	1	
Second-year	17(20.2)	67(79.7)	2.07 (0.91–4.72)	1.83 (0.5–6.65)	0.36
Third-year	56(48.3)	60(51.7)	7.63 (3.70–15.75)	2.02 (0.57–7.23)	0.28
Fourth-year	52(61.9)	32(38.1)	13.29 (6.18–28.58)	2.23 (0.57–8.70)	0.25
**Religion**					
Christian	113(39.6)	172(60.4)	1	1	
Muslim	17(23.3)	56(76.7)	0.46 (0.26–0.83)	0.56 (0.22–1.46)	0.24
Traditionalist	1(100)	0			
No religion	5(19.2)	21(80.7)	0.36 (0.13–0.99)	0.11 (0.02–0.55)	<0.01
**Ethnicity**					
Akan	56(37.8)	92(62.2)	1		
Ewe	40(33.6)	79(66.4)	0.83 (0.50–1.38)		
Ga-Adangbe	21(38.2)	34(61.8)	1.02 (0.53–1.92)		
Others	19(30.2)	44(69.9)	0.71 (0.37–1.34)		
**Faculty**					
Nursing & Midwifery	76(37.6)	126(62.4)	1		
Allied health Sciences	24(31.6)	52(68.4)	0.77 (0.43–1.34)		
Basic and Biomedical Sciences	7(100.0)	0(0.0)			
Medicine	27(31.4)	59(68.6)	0.76 (0.44–1.29)		
Pharmacy	2(14.3)	12(85.7)	0.28 (0.06–1.27)		
**Known someone with breast cancer**				
Yes	79(56.0)	62(43.9)	1	1	
No	57(39.6)	87(60.4)	0.51 (0.32–0.82)	1.27 (0.60–2.70)	0.53
**Risk perception (N = 281)**					
At risk	94(88.7)	12(11.3)	1	1	
Not at risk	34(26.4)	95(73.6)	0.05 (0.02–0.09)	0.04 (0.02–0.09)	<0.001
Do not know	7(15.2)	39(84.8)	0.02 (0.008–0.06)	0.02 (0.005–0.57)	<0.001
**Family history of breast cancer**					
Yes	39(70.9)	16(29.1)	1	1	
No	97(29.4)	233(70.6)	0.17 (0.09–0.32)	0.56 (0.21–1.45)	0.23
**Physical activity**					
Active	125(36.1)	221(63.8)	1		
Inactive	11(28.2)	28(71.8)	0.69 (0.33–1.44)		
**Current Alcohol Use**					
Yes	37(48.1)	40(51.9)	1	1	
No	99(32.1)	209(67.9)	0.51 (0.3101.85)	0.47 (0.19–1.12)	0.09
Current Contraception use*					
Yes	21(41.2)	30(58.8)	1		
No	115(34.4)	219(65.6)	0.75 (0.41–1.36)		

*Pills or injectables; cOR: Crude Odds Ratio; aOR: Adjusted Odds Ratio.

## Discussion

This study though has demonstrated considerable awareness about the existence of breast cancer, insufficient knowledge, and misconceptions regarding its risk factors and causes and disease presentation also existed among participants. Less than three-quarters of our study participants had heard about breast cancer. This is unexpectedly far lower than the 100% observed in medical students in Harar, Ethiopia [23], 98.7% among University of Ibadan female students [24], 95% previously reported among female students of Faculty of Health and Medical Sciences in Ghana [25], and 88.1% among Teacher Training college students in Cameroon [26]. The difference in the awareness rate found in this study and that of the aforementioned studies cannot directly be explained. However, breast cancer has been adopted in the curriculum of the first two aforementioned studies in an attempt to create awareness among students and might have had a positive impact on the students’ awareness about the disease. Ours finding further show unsatisfactory levels of awareness and understanding of breast cancer risk factors and disease presentation. More than one-third of participants were not recognised increasing age, nulliparity, obesity, and early menstruation and late menopause as potential risk factors for breast cancer, while 5% did not know any risk factor for breast cancer. This finding confirms reports from previous studies in Ethiopia [27], Nigeria [28], Egypt [29], and Angola [30] where general knowledge and risk factors of breast cancer were found to be low among female college students. Knowledge gaps have also been identified among the general population elsewhere [31, 32]. About one-third of our respondents held the belief that putting money in the brassiere can result in breast cancer. This is in line with other studies among women in the general population [33, 34] as well as university students [24] that suggest that women still have misperceptions about the cause of breast cancer with some attributing it to the spiritual origins. Additionally, as reported by previous studies elsewhere, [23, 26] awareness of other clinical manifestations of breast cancer other than breast lump was worryingly low. These existing knowledge gaps and misconceptions may impact health-seeking behaviours and uptake of breast screening resulting in late diagnosis which in turn may lead to complications and death. Thus, the need for health education programmes aiming to increase awareness about the causes, risk factors and clinical presentation of breast cancer is warranted.

Predictors of breast cancer risk are varied, including individual lifestyle, reproductive status, and genetics. This study attempted to identify some of these risk factors among the students. First menstruation at an early age (early menarche) is known to be associated with increased levels of endogenous hormones (estrogen and progesterone) in a woman’s lifetime which increases the risk of breast cancer [17]. In this study, about 13% of participants had their menarche at an age younger than 12 years. In the Lublin region of Poland, menarche occurred at 11 years in only about 3% of women attending screening a programme [35].

Among the most extensively researched risk factors of breast cancer is the use of exogenous hormones in the form of oral contraceptives and hormone replacement therapy (HRT) [36]. Lina et al. [37] in their study to assess the association between the use of hormonal contraception and the risk of invasive breast cancer among 1.8 million Danish women demonstrated that current and recent users of hormonal contraception had 20% increased risk of breast cancer compared with those who had never used hormonal contraception. We found in this study that, about 13% of women were current users of oral pills/injectables. None of them was however on HRT.

A positive family history of breast cancer in a first-degree relative is the most commonly known risk factor for the disease [38]. Women with a family history of breast cancer in a mother or sister have up to a 3-fold increase in the risk of developing breast cancer [38]. In this present study, about 14% of the participants had a positive family history of breast cancer, among whom 4% occurred in first degree relatives (mother and sister), and hence have increased risk of the disease. Our result is higher than that reported in the previous study in Ghana, where 1.4% of women had a first-degree family history of breast cancer [8]. and another study among university students in Ajman, UAE, where a positive family history of breast cancer was found in 9% of students and about 1% with the first-degree relative affected with breast cancer [39]. Researchers have devoted much attention to understanding the role that genes play in the development of breast cancer. This has helped in recognizing that some women could be at increased risk as a result of inherited predisposition. It must be acknowledged however that other than genes, families also share other factors such as cultural background and environmental exposures, which are themselves potential predictors of breast cancer [39].

The relationship between alcohol consumption and an increased risk of developing breast cancer has been the subject of many studies. Compared with non-alcohol drinkers, women who drink even in small amounts, have increased risk of breast cancer. The risk increases with an increased amount of alcohol consumed per day [40]. In many traditional African societies including Ghana, alcohol consumption is not common among females and the youth and people usually frown on alcohol intoxication [41]. The prevalence of alcohol consumption in African women varies from 1% in Malawi to 30% in Burkina Faso with about 81% of women in Africa reporting lifetime abstinence [42]. The prevalence of current alcohol consumption (20%) among female undergraduates found in this study is similar to levels (19.6%) reported among women in the WHO African regions in 2012 [43]. Martinize et al. [42] reported that about two-thirds of Ghanaian women abstain from alcohol in their lifetime. Kofi Adesi Kyei and colleagues found in their previous 5-year retrospective review to identify predominant lifestyle risk factors of breast cancer among Ghanaian women that alcohol contributed to about 19% to the disease with respect to preventable risk factors [44] and concluded that alcohol is not the most important preventable risk factor for breast cancer in Ghana. However, having 2 in 10 future female health professionals drinking alcohol is significant enough to warrant public health action due to the health consequences of alcohol on women.

Many studies have reported an inverse relationship between regular physical activity and breast cancer risk. Several biologic mechanisms have supported the protective effect of physical activity on breast cancer which includes effect on immune, endogenous sex steroid hormone production, and antioxidant system [44–46]. In this present study, 1 in 10 women was not physically active. Of those who were, the majority engaged in brisk walking. Comparing the most active and least active women, most studies estimate the risk reduction of breast cancer to be between 20 and 40% and recognise the dose-response relationship with risk increase levels of risk reduction [47–49]. Several mechanisms might be the cause of this inverse association with physical activity. Increased levels of activity are known to reduce body weight, thus reducing the risk of breast cancer. Physical activity may also influence the production, metabolism, and excretion of endogenous hormones that can result in lower levels of bioactive oestrogen, insulin, and other growth factors [50].

Accurate and early diagnosis of breast cancer depends mainly on the "opportunist approach". Given the challenges faced by resource-limited countries, improving breast cancer awareness and the application of screening methods remains a practical option for early detection and treatment of breast cancer. Similar to reports from previous studies done in different parts of the world including Ghana at different times, the performance of breast cancer examination is generally low [51–56]. Notably in this study, less than half of the participants performed BSE, 10% had CBE, and an even lower percentage (2.3%) had Mammography. This is a worrying phenomenon owing to the fact that breast cancer is increasingly becoming common in this part of the world, hence women and young ladies need to frequently subject themselves to screening for early detection and treatment to avoid complications and death. The low coverage of breast cancer screening among our study population could perhaps be explained by their young age as breast cancer has been known to be common among older women.

While the effectiveness of BSE to detect breast malignant tumour remains debatable, its importance in breast self-awareness creation in resource-limited countries with non-existent population screening programmes cannot be overemphasis, thus deserves consideration. The level of BSE practice in this study is, however, higher than that reported among over 10,000 undergraduate students from 24 countries across Africa, Asia and America. (9.1%) [57], and that of other studies among students in Ethiopia (39.4%), Cameroon (3%), Libya (23.5%) [53, 58, 59]. The rising trends in the incidence of breast cancer in Africa may be explained by the lack of health consciousness of young women to examine themselves for the timely identification of any breast abnormality. In this study, lack of skills, no family history of breast cancer and pessimism about the risk of the disease were the most important reasons for not performing BSE. This is a worrying finding since these are future healthcare professionals who are required to educate others in the community about the disease and the need for primary prevention. University students are among the well-informed group of women in Ghana. Their lack of skills in performing breast screening and the need for periodic screening, therefore, is indicative of a greater lack of skills and awareness among the general population of less-educated women. Similar, in Ethiopia, being healthy and lack of knowledge were the overriding factors given [53]. In a previous study among Presbyterian university college students in Ghana, varied reasons including, lack of time, forgetfulness, procrastination, and fear were the reasons for not performing BSE [25]. The social media could be an important tool that can be harnessed to educate women on the need for regular and correct practice of BSE since it remained the most important source of education for women who performed BSE in this study.

Expectedly, this study demonstrates that risk perception, an important component of behavioural change paradigm, is a sufficient enough variable that is capable of affecting women’s breast cancer screening behaviour. Women who did not believe to be vulnerable to breast cancer and those who did not know their risk status were less likely to practice BSE regularly compared to those who were pessimistic about their risk of the disease. Our result confirms the assertion of the Health Belief model that women who with a higher risk for breast cancer, perceive breast cancer as a serious threat, have a lower perception barrier, and who hold a higher perception of the benefits are more likely to perform regular BSE [60]. Erbil and Bolukbas also suggest that women’s health beliefs and attitudes remain the predominant factors that influence whether or not they will get themselves screened for breast cancer [61]. Among Korean women, those with lower perceived comparative risk were more likely to have no intention of getting a mammogram [62]. Further, this study demonstrated that who were not affiliated to any religion were less likely to examine their breast regularly compared to their counterparts who belonged to the Christianity religion perhaps due to exposure to breast cancer information in churches.

It is interesting to note that other risk variables such as physical activity, Alcohol consumption, and contraception use, and positive family history did not influence breast BSE in this present study. This is a worrying finding because women with risk factors are expected to perform regular breast examinations to detect the disease at its early stage to prevent complications and death. It can be suggested that university women do not practice BSE even though they have some risk factors of breast cancer.

There are three limitations of our study worth mentioning. First, the findings cannot be generalised beyond the study population since they are young and well-educated women. University students are not representative of young adults in general, and the risk perception, risk factors, and breast cancer screening practice may differ from that of the general population. Secondary, all data were self-reported with no objective measures to assess the accuracy of these reports. Lastly, the instrument used was not tested for its validity, however, questions used were taken from validated tools from previous studies at other settings. Nevertheless, the results of this study provide some understanding regarding perceived risk and the practice of breast screening among future health professionals, which can be useful for directed health promotion and education.

## Conclusion

The research concludes that the awareness of breast cancer and its causes, risk factors, and disease manifestation was generally unsatisfactorily low. Additionally, even though some students possess some important risk factors of the disease, the practice of BSE coverage which was influenced by risk perception and religion was low. Improved methods of risk communication are recommended to ensure that women have appropriate risk information to make informed choices about risk management options and preventative interventions. Social media can also be a reliable tool for health education on breast cancer.

## Supporting information

S1 Data Anonymised data set used for this study. (XLS)

S1 Questionnaire Assessment of breast cancer risk factors and screening practices among university female students, Ghana. (DOCX)
