# Supplementary material for: Breast cancer awareness, risk factors and screening practices among future health professionals in Ghana: A cross-sectional study
Source: PLoS One. 2021 Jun 24;16(6):e0253373. doi: 10.1371/journal.pone.0253373 (PMC8224936; doi:10.1371/journal.pone.0253373)
Supplement: S1 Questionnaire — (DOCX) [file pone.0253373.s002.docx]

**QUESTIONNAIRE**

**ASSESSMENT OF BREAST CANCER RISK FACTORS AND SCREENING PRACTICES AMONG UNIVERSITY FEMALE STUDENTS, GHANA**

**No. [ ] Please cycle the number that corresponds to the respondent’s answer**

| **Demographic characteristics of respondent** | | |
| --- | --- | --- |
| **No.** | **Variable** | **Response** |
| 01 | Program offering | 1. Nursing 2. Midwifery 3. Medicine 2. Medilab 5. Pharmacy   6. Others………………………………… |
| 02 | Year of study | 1. Level 100 2. Level 200 3. Level 300 4. Level 400 |
| 03 | Type of student | 1. Regular 2. Top up |
| 04 | Age (years) | _________ |
| 05 | Marital status | 1. Ever Married 2. Currently married 3. Never married |
| 06 | Religious affiliation | 1. Christian 2. Muslim 3. Traditionalist 4. No religion |
| 07 | Ethnicity | 1. Akan 2. Ewe 3. Ga Adangbe 2. Others_______________ |
| **Awareness of breast cancer** | | |
| 08 | Have you heard of breast cancer before? | 1. Yes 2. No {skip to 24} |
| 09 | If yes source of information | 1. Print media 2. Social media 3. Electronic media 4. Friends and relatives 5. Teacher   6. Health facility/worker |
| 10 | Have you ever seen  /known someone with breast cancer? | 1. Yes 2. No 3. Can’t remember |
| 11 | Which breast cancer screening method are you aware of? | 1. BSE 2. CBE 2. Mammography 3. None |
| 12 | What are(is) the risk factors for breast cancer (mention all you know) | 1. Genetics **2.** Drugs **3.** Radiation **4**. Aging   **5**. Female sex **6.** Putting money in brassiere  **7**. Lifestly **8**. Nulliparity **9.** Early menses/late menopause **10**.Obesity 11. Family history of breast cancer **12**. Other…………………………….**13.** Don’t know |
| 13 | Which sign/symptom of breast cancer do you know? (mention all) | 1. Lump in breast **2**. Nipple discharge **3.** Swollen nipple **4**. Ulcerated breast **5**. Inverted nipple **6**. Pain in breast **7**. Redness of breast **8**. Nipple itch **9**. Lymph node in armpit **10**. Pulling of nipple   **11**. Others…………………….. 12. Don’t know |
| **Breast cancer screening practices** | | |
| 14 | Which of the screening methods have you ever done? | 1. BSE 2. CBE 3. Mammography   4. Never done breast screening (answer 15 and skip to 20) |
| 15 | If never done breast screening before, do you have any intension of doing it? | 1. Yes 2. No |
| 16 | If done BSE before at what age did you start? | ___________ |
| 17 | Who taught of how to do BSE | 1. Mother **2.** Teacher **3.** Media **4**. Friend   **5**. Other relative **6.** Never taught |
| 18 | How often do you do BSE? | 1. Daily 2. Weekly 3. Monthly 4. yearly 5. Any time I feel like doing it 6. Any time I suspect something 7. Can’t remember   8. Other____________ |
| 19 | How do you do BSE? | 1. Lying down 2. Standing up 3. Setting down |
| 20 | If not done BSE before what is the reason? | 1. Don’t know how to do it **2**. Not necessary **3.** No breast cancer in my family **4.** Not at risk of breast cancer **5**. Do not have time 6. No reason **7**. Other___________ |
| 21 | At what age should one start BSE | ____________ |
| 22 | Do you perceive yourself of being at risk of breast cancer? | 1. Yes {skip to 24) 2. No 3. Don’t know |
| 23 | If no, why | 1. Take good care of myself 2. I do regular breast examination 3. No history of breast cancer in my family 4. Other____________________________ |
| **Risk factors of breast cancer** | | |
| 24 | At what age did you have your first menses? | _______________ |
| 25 | At what age did you have your first sexual intercourse? | 1. ____________ 2. Can’t remember 3. Never had sex |
| 25 | Have you been pregnant before? | 1. Yes 2. No |
| 26 | Have you had induced abortion before? | 1. Yes 2. No |
| 27 | Have you had a miscarriage before? | 1. Yes 2. No 3. Can’t remember |
| 28 | How many pregnancies have you had? | 1. __________ 2. 2. Never been pregnant |
| 29 | How many children do you have? | 1. _____________   2. Never given birth |
| 30 | At what age did you have your first child? | _____________ |
| 31 | Have you ever used oral contraception or injectable before? | 1. yes 2. No |
| 32 | Do you currently use oral contraception or injectable? | 1. Yes 2. No |
| 33 | How long have you been using oral contraception or injectable? | _____________ |
| 34 | Does any of your relatives ever had breast cancer? | 1. Yes 2. No |
| 35 | If yes who? | 1. Sister 2. Mother 3. Grandmother 4. Auntie   5. Other relative |
| 36 | Do you do anything to keep fit? | 1. Yes 2. No |
| 37 | If yes what physical activity do you do? | 1. Walking 2. Jogging 3. Sports 2. 4. Other_____________________ |
| 38 | How often do you do this activity | _________________ |
| 39 | Do you currently drink alcohol? | 1. Yes 2. No |
| 40 | How many bottles of alcohol can you consume at t sitting? | ________________ |
| 41 | How often do you drink alcohol? | 1. Daily 2. Every other day 3. Every three days   4. Once weekly 5. Bi-weekly 6. Monthly 7. Anytime  8. other___________________ |
|  | Have you been diagnosed of breast cancer before? | 1. Yes 2. No |
